# Cardiometabolic Risk: Characteristics of the Intestinal Microbiome and the Role of Polyphenols

**DOI:** 10.3390/ijms241813757

**Published:** 2023-09-06

**Authors:** Ioana Mariana Haș, Delia Mirela Tit, Simona Gabriela Bungau, Flavia Maria Pavel, Bernadette-Emoke Teleky, Dan Cristian Vodnar, Cosmin Mihai Vesa

**Affiliations:** 1 Doctoral School of Biological and Biomedical Sciences, University of Oradea, 410087 Oradea, Romania; ioanahas@gmail.com (I.M.H.); flavia.m.pavel@gmail.com (F.M.P.); v_cosmin_15@yahoo.com (C.M.V.); 2 Department of Pharmacy, Faculty of Medicine and Pharmacy, University of Oradea, 410028 Oradea, Romania; 3 Institute of Life Sciences, University of Agricultural Sciences and Veterinary Medicine, 400372 Cluj-Napoca, Romania; bernadette.teleky@usamvcluj.ro (B.-E.T.); dan.vodnar@usamvcluj.ro (D.C.V.); 4 Department of Food Science and Technology, University of Agricultural Sciences and Veterinary Medicine, 400372 Cluj-Napoca, Romania; 5 Department of Preclinical Disciplines, Faculty of Medicine and Pharmacy, University of Oradea, 410073 Oradea, Romania

**Keywords:** cardiometabolic risk, gut microbiota, microbiome, polyphenols, dysbiosis, prebiotics

## Abstract

Cardiometabolic diseases like hypertension, type 2 diabetes mellitus, atherosclerosis, and obesity have been associated with changes in the gut microbiota structure, or dysbiosis. The beneficial effect of polyphenols on reducing the incidence of this chronic disease has been confirmed by numerous studies. Polyphenols are primarily known for their anti-inflammatory and antioxidant properties, but they can also modify the gut microbiota. According to recent research, polyphenols positively influence the gut microbiota, which regulates metabolic responses and reduces systemic inflammation. This review emphasizes the prebiotic role of polyphenols and their impact on specific gut microbiota components in patients at cardiometabolic risk. It also analyzes the most recent research on the positive effects of polyphenols on cardiometabolic health. While numerous in vitro and in vivo studies have shown the interaction involving polyphenols and gut microbiota, additional clinical investigations are required to assess this effect in people.

## 1. Introduction

According to World Health Organization (WHO) data, 74% of deaths worldwide are caused by a non-transmissible disease. Cardiovascular diseases (CVD) and diabetes (diabetes mellitus) are two of the top three non-transmissible diseases, causing 19.9 million deaths [1]. On the other hand, globally, we are facing an obesity epidemic. According to the WHO estimates for 2016, 52% of the world’s adult population was overweight or obese. Unfortunately, the situation also worries children and teenagers, the future adults [2]. Obesity is one of the most critical risk factors for CVD and is associated with diabetes, high blood pressure, and sleep apnea [3]. Even modest excess weight has been correlated with a significant increase in the risk of cardiovascular mortality [4]. At the same time, reducing body weight by only 5–10% reduces cardiometabolic risk by improving insulin sensitivity, lipid profile, and endothelial function. It positively influences the thrombotic and inflammatory processes [5]. The causes of the global epidemic of coronary heart disease (CHD) appear to be essentially the same anywhere in the world. In the INTERHEART study, one of the most essential and extensive studies in cardiology, a cross-sectional study conducted on 27,000 subjects in 52 countries on all continents identified nine risk factors responsible for over 90% of the risk of myocardial infarction: smoking, abnormal lipid levels, high blood pressure, diabetes, abdominal obesity, diet, lack of physical activity, alcohol consumption, and psychosocial stressors [6].

Regarding the prevention of CVD and diabetes, an efficient approach is the assessment of cardiometabolic risk, which helps in both primary and secondary prevention, allowing therapeutic intervention to prevent disease progression and complications [7,8]. The term “cardiometabolic risk” is an “umbrella” term coined by the American Diabetes Association and refers to a group of risk factors and metabolic markers based on which people at high risk of developing CVD, vascular events, and diabetes will be identified [9,10]. It became necessary in the more complex approach and assessment of cardiometabolic risk, completing the concept of metabolic syndrome. This concept includes many factors contributing to the increase in CVD and metabolic risk [11].

Metabolic syndrome (MS) is a complex metabolic disorder with insulin resistance, compensatory hyperinsulinism, and abdominal obesity at the center of the pathophysiology [12]. In 1998, the MS was first defined by the WHO, with the definition being set as the diagnostic criteria, establishing as a mandatory criterion the presence of insulin resistance [12]. In 2001, NCEP-ATP III proposed a new set of diagnostic criteria without a mandatory criterion [12]. The most recent set of criteria is the one issued by the International Diabetes Federation in 2005, imposing as a mandatory criterion the presence of obesity [13]. The standard criteria for identifying people with metabolic syndrome are abdominal circumference, fasting blood glucose, triglyceride and HDL-cholesterol levels, and blood pressure. A separate criterion, suggested by the WHO, is the value of albuminuria [14]. Although the presence of MS increases two times the risk of cardiovascular events and cardiovascular mortality, 1.5 times the risk of mortality from any cause, and up to five times the risk of developing diabetes, it cannot estimate the overall cardiovascular risk because it provides only a part of the complete picture and does not take into account the significant traditional risk factors [15,16,17]. In this sense, so-called cardiovascular risk calculators have developed over time, such as Framingham, Reynolds, UKPDS, PROCAM, SCORE, and others [18,19,20,21,22,23,24,25]. Of course, each algorithm has advantages and limitations. Some have specificity in certain populations: Framingham is based on a study conducted on the American population, while SCORE is based on the results of 12 cohort studies conducted on European subjects. Some provide predictions regarding the risk of any coronary heart disease event, while others focus on estimating total fatal cardiovascular risk (SCORE). In some algorithms, we encounter age-related limitations (SCORE); others are specific to certain types of patients, e.g., UKPFD for people with diabetes. But in general, the risk in patients with risk factors such as obesity and insulin resistance is underestimated [10,20,26,27,28,29]. In 2006, Després and Lemieux showed, through a practical exercise, that meeting the clinical criteria for the diagnosis of MS in a person does not necessarily entail an absolute risk of CVD, emphasizing the importance of assessing traditional risk factors [10]. They defined cardiometabolic risk as the result of metabolic syndrome in combination with the presence of conventional risk factors such as blood pressure, fasting blood glucose, lipid profile, age, sex, smoking, and family history [10].

There is a causal association between cardiometabolic diseases and gut microbiota [27]. The gut microbiota represents a significant element in terms of the state of human health. The state of intestinal dysbiosis contributes to the development of many intestinal and extraintestinal diseases, including CVD and diabetes [28,30,31]. Recent studies have shown that polyphenols positively affect the gut microbiota, resulting in the regulation of metabolic responses and the decrease of systemic inflammation. Cardiometabolic protective properties of polyphenolic compounds, through different mechanisms, have been proven in numerous studies, with their dietary consumption associated with anti-inflammatory and antioxidant effects and the improvement of endothelial function, glycemic, and lipid profiles [32,33]. Moreover, the prebiotic potential of polyphenols has been demonstrated. At the end of 2016, the International Scientific Association for Probiotics and Prebiotics updated the definition of prebiotics, more precisely, “a substrate that is selectively used by the host microorganism that confers a health benefit”, expanding their list to include phenolic and phytochemical substances [34].

This review summarizes the current knowledge regarding the beneficial effects of polyphenols on cardiometabolic health. It also reviews recent experimental and clinical data for exploring their prebiotic role and the impact on specific aspects of the gut microbiota of patients at cardiometabolic risk.

## 2. Methodological Strategies

**Figure 1:**
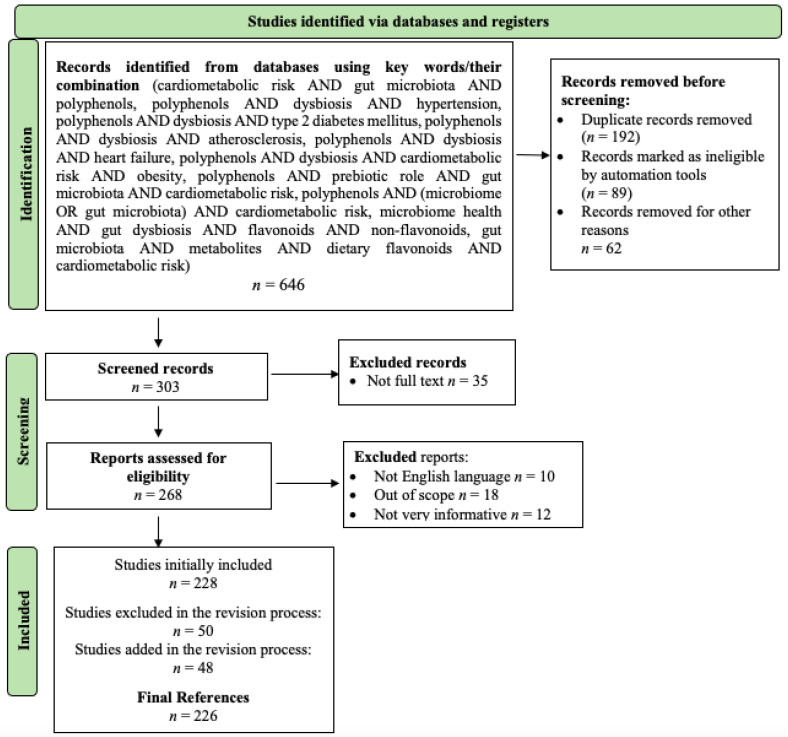
Literature selection depicted in a PRISMA 2020 flow diagram.

The present paper evaluated scientific publications exploring the impact of polyphenols on specific gut microbiota components in patients at cardiometabolic risk. This assessment is based on an extensive literature search using reputable databases such as Scopus, PubMed, SpringerLink, Nature, and ScienceDirect. The selection process for identifying relevant scientific publications involves a well-defined algorithm that leverages Boolean logic operators, precisely AND and OR. These operators are integrated into the criteria established in the PRISMA flowchart, a recognized tool for systematically evaluating and filtering out ineligible results. Figure 1 illustrates the logical sequence of operations employed to determine the final resource set in this narrative review. This process aligns with the guidance provided by Page et al. in their recommendations for the PRISMA flowchart algorithm [34]. Before the screening stage, publications not in English, those lacking substantial, informative content, or those not falling within the category of articles or books were excluded from consideration. The reliability of the information presented in this paper was ensured by 226 bibliographic sources spanning 1993 to 2023, which were thoroughly evaluated and cited.

## 3. Particular Aspects of the Gut Microbiota of Patients at Cardiometabolic Risk

The gut microbiota, a collection of bacteria, eukaryotes, and archaea that colonize the digestive tract, has evolved with the host, creating a symbiotic relationship between the two “parts” [35]. It is estimated that the ratio between the number of bacterial cells, dominated by colon bacteria, and human cells is approximately 1:1 [36].

The relationship between microorganisms and the host plays a very important role in human health and disease. The host’s lifestyle, age, and environmental factors greatly influence the diversity of the gut microbiota. Diet is one of the most important factors in modulating the gut microbiota [37]. The last two decades have been characterized by a great interest in scientific research of the gut microbiota, with intestinal dysbiosis being associated with a multitude of diseases, from intestinal diseases to metabolic, cardiovascular, and neurological diseases, allergies, and even some forms of cancer [38,39,40,41,42,43,44].

The human gut microbiota is dominated by two prominent families, namely *Bacteroidetes* and *Firmicutes*, and in smaller proportions are found *Proteobacteria*, *Verrucomicrobia*, *Actinobacteria*, and *Fusobacteria* [45]. It plays a key role in modulating host immunity, metabolic processes, digestion, maintaining the structural integrity of the intestinal mucosa barrier, and maintaining homeostasis [46]. An unbalanced, stressful lifestyle, inadequate eating habits, climate, pollution level, hormonal changes, and antibiotic treatment are some of the most important factors determining intestinal dysbiosis onset [47,48]. Identifying the balanced composition of the gut microbiota and the particular aspects specific to certain diseases is essential [49,50].

The main ways in which the gut microbiota influences the cardiometabolic health of the host are correlated with the composition and bacterial diversity, the immune response to bacterial components (lipopolysaccharides, lipoproteins, and peptidoglycans) translated by inflammation, and the involvement of bacterial metabolites, especially short-chain fatty acids (SCFA) and trimethylamine N-oxide (TMAO), respectively, in the host metabolism [51,52,53].

### 3.1. Bacterial Composition and Diversity

Several human or animal studies have shown that obesity, diabetes, and CVD are associated with a low *Bacteroidetes*/*Firmicutes* ratio and low microbial diversity. The latter also correlated with increased adiposity, insulin resistance, an altered lipid profile, and low-degree inflammation [54,55,56,57]. Low-degree systemic inflammation, or meta-inflammation, characterized by elevated C-reactive protein levels, is correlated with the pathogenesis of metabolic and cardiovascular diseases and various types of cancer [58]. 

*Faecalibacterium prausnitzii*, a gram-positive anaerobic bacterium, has been shown to be one of the species with anti-inflammatory potential, being found in small amounts in individuals with obesity, diabetes, or CVD [59,60,61]. *Akkermansia muciniphila*, another bacterial species with anti-inflammatory potential and a gram-negative anaerobic bacterium associated with metabolic health, has been found in small amounts in both obese and diabetic subjects [62,63]. *Roseburia intestinalis*, a gram-positive anaerobic bacterium and one of the most important butyrate-producing bacteria, has been shown to have a low abundance in subjects with diabetes and CVD [64,65]. Other species with low presence in obese subjects, diabetes, or CVD were as follows: *Methenobrevibacter smithii*, *Lactobacillus casei*, *Lactobacillus paracasei*, *Lactobacillus plantarum*, and *Bacteroides thetaiotaomicron*, *Bacteroides fragilis* [56,66,67]. On the other hand, an increased abundance was seen for species such as *Lactobacillus reuteri*, *Ruminococcus gnavus*, *Ruminococcus torques*, *Ruminococcus bromii*, *Clostridium clostridioforme*, *Escherichia coli*, *Eubacterium biforme*, and *Eubacterium ventriosum* [56,66,67,68,69].

A deficient presence of *Bacteroides fragilis*, *Lactobacillus casei*, *Lactobacillus plantarum*, and *Akkermansia muciniphila* determines the “installation” of the permeable intestine by affecting the intestinal barrier function, and implicitly endotoxemia is determined by the translocation of bacterial products in the host’s circulation [65]. Reduced microbial diversity has been correlated with the presence of several species with inflammatory potential [54,70]. Dysbiosis is practically a triggering factor of the inflammatory process through various mechanisms, among which the excessive production of C-reactive protein, some pro-inflammatory interleukins or tumor necrosis factor, reactive oxygen species, or increased expression of the vascular endothelial adhesion molecule 1 [66]. Some specific aspects regarding the diversity of gut microbiota in people with cardiometabolic pathologies reported in previous studies are presented in Table 1.

### 3.2. The Immune Response to Bacterial Components

For a better understanding of the mechanisms by which the state of the gut microbiota is involved in the pathogenesis of cardiometabolic diseases, it is essential to understand the importance of the bacterial components and microbial metabolites.

Lipopolysaccharides (LPS) are some of the most important bacterial components. They are major components of the outer membrane of gram-negative bacteria and endotoxins responsible for activating systemic inflammation through TLR4 receptors, a condition associated with the onset of many diseases, including obesity, diabetes, and CVD [74,75]. They are found in the bloodstream in minimal quantities and do not trigger an immune response. LPS enters the systemic circulation without an intact intestinal barrier, causing endotoxemia [76]. Metabolic endotoxemia seems to be induced primarily by an unbalanced diet rich in saturated fats, chronic alcohol consumption, and aging [77,78,79].

Several studies have investigated the correlation between metabolic endotoxemia and the development of cardiometabolic diseases. Some of the study findings: metabolic endotoxemia appears to precede the development of diabetes; subjects with diabetes showed, in the systemic circulation, LPS values three-quarters higher than the control group; higher levels of endotoxins in the systemic circulation were correlated with a four-times higher risk of atherosclerosis; high-fat meals are correlated with an increase in endotoxin concentrations; so it is suggested that plasma levels of LPS be a predictive biomarker of this condition [80,81,82].

### 3.3. Involvement of Bacterial Metabolites

Another mechanism by which the gut microbiota can impact the host metabolism is the production of bacterial metabolites, some of which are positively correlated with cardiometabolic health (short-chain fatty acids), others negatively correlated (trimethylamine N-oxide) [83]. Short-chain fatty acids (SCFA) are bacterial metabolites resulting from the fermentation of dietary fibers that reach the colon without being digested or absorbed in the small intestine [84]. The most important SCFAs are butyrate, acetate, and propionate [84]. They play several roles: they are a source of energy, help regulate glucose and lipid metabolism, regulate the immune system and inflammation, defend the intestinal barrier, and regulate appetite [85].

Several studies have evaluated the importance of a high-fiber diet in patients with obesity, diabetes, or CVD in order to increase the concentration of SCFA by increasing the abundance of SCFA-producing bacteria [86,87,88]. *Faecalibacterium prausnitzii*, *Eubacterium rectale*, *Eubacterium hallii*, *Roseburia faecis*, and *Roseburia intestinalis* seem to be the main producers of butyrate [64,88]. The results showed that SCFA are able to positively influence certain risk factors associated with cardiometabolic diseases, such as lipids, carbohydrate profiles, and body weight [86,87,89].

According to several studies, trimethylamine N-oxide (TMAO), the liver oxidation product of the microbial metabolite trimethylamine, is a promoter of cardiovascular diseases [83]. A metabolite that could be useful as a CVD biomarker is trimethylamine. It is generated in the largest proportion in the intestine, deriving from carnitine and choline, nutrients found primarily in red meat, fish, dairy, and eggs [90].

The association of TMAO with undesirable clinical consequences, pro-atherogenic and pro-thrombotic effects, increased risk of major cardiovascular events, and influence on glycemic control in patients with diabetes has been shown in several studies performed on a large number of patients [52,53,90,91,92]. There are correlations between the composition of the microbiota and the production of TMAO, noting that a high-fat diet is correlated with the low presence of *Bacteroidetes* and high TMAO values. In contrast, a low-fat diet correlates with the high presence of *Bacteroidetes* and increased TMAO values [93].

## 4. Beneficial Effects of the Polyphenols Action in Terms of Cardiometabolic Health

In recent decades, the interest in researching polyphenols and, implicitly, their health benefits have been found as impressive. The first evidence of this comes from studies conducted in the 1960s [94]. Since then, several studies have brought to attention the beneficial effects of dietary polyphenol consumption correlated with a reduced risk of CVD, diabetes, neurodegenerative and autoimmune diseases, certain types of cancer, and allergies [95,96,97,98,99].

**Figure 2:**
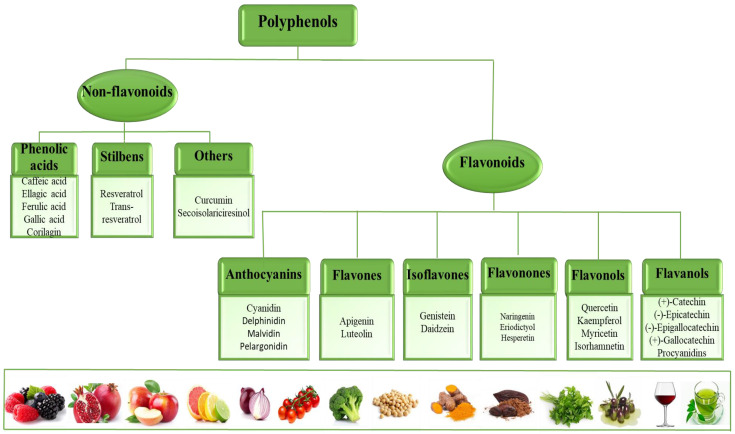
Dietary polyphenol subclasses with roles in cardiometabolic health.

Polyphenols are natural compounds in foods available daily in the human diet, namely fruits, vegetables, whole grains, and beverages (i.e., tea, chocolate, and wine) [100,101,102]. More than 8000 phenolic structures have been identified [100], with phenolic compounds practically being secondary metabolites of plants with at least two aromatic nuclei and one or more hydroxyl groups [101]. Based on their chemical structure, polyphenols were classified into two major classes, namely: non-flavonoids, subclassified into phenolic acids, stilbenes, and lignans, respectively; flavonoids, subclassified into anthocyanins, flavonols, flavones, isoflavones, flavanones, and flavanols (Figure 2) [103].

**Figure 3:**
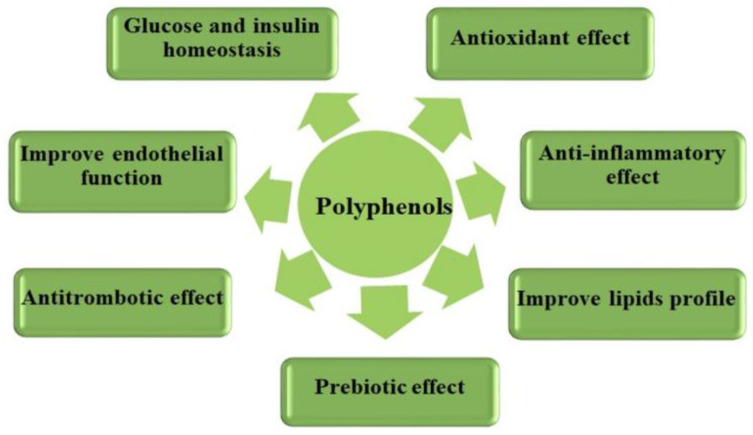
Polyphenols effects and cardiometabolic risks.

Numerous interventional, epidemiological, prospective, and meta-analyses have shown that regular consumption of foods high in polyphenols determines cardioprotective effects, decreased cardiovascular morbidity and mortality, and the prevention of diabetes [95,104,105,106,107,108,109,110,111,112,113,114] through antioxidant, anti-inflammatory, and antithrombotic action, improvement of endothelial function and lipid profile, involvement in glucose and insulin homeostasis, and prebiotic effect, as summarized in Figure 3.

### 4.1. Antioxidant Effect

Oxidative stress is defined simply as an imbalance between the production of free radicals in cells and tissues and the body’s antioxidant defense capacity. Scientific data show that this imbalance is essential in the pathogenesis of several chronic diseases, including cardiovascular diseases [115].

Diet is a significant factor in the pathogenesis of cardiometabolic diseases. While fruits, vegetabless and red wine have a protective effect by reducing oxidative stress, a high-fat diet induces it. The high content of polyphenols in fruits and vegetables is the most important factor in reducing oxidative stress [116]. The antioxidant action of polyphenols is mainly due to their ability to capture free radicals by transferring the H atom or an electron from the active hydroxyl group(s) in the annular structures of phenolic compounds to the free radical. On the other hand, it can also act by chelating metals that catalyze the formation of radicals (copper, iron, and manganese) [95]. 

In addition to the direct mechanism of free radical scavenging, polyphenols have the ability to inhibit the expression of enzymes involved in the production of free radicals, such as xanthin oxide, lipoxygenase, cyclooxygenase, myeloperoxidase, or nicotinamide adenine dinucleotide phosphate oxidase. On the other hand, they induce enzymes with antioxidant activity, such as glutathione peroxidase, superoxide dismutase, and catalase [117].

A recent theory states that beyond the direct or indirect mechanisms mentioned above, the antioxidant action of polyphenols would result from their ability to modulate signaling pathways at the intracellular level, regulating the activity of protein and lipid kinases [118].

The antioxidant potential is influenced by the number and position of hydroxyl groups in the structure of polyphenols. For example, polyphenols with hydroxyl groups in the ortho and/or para positions on the benzoic ring have a higher antioxidant capacity than polyphenols with hydroxyl groups in other positions [119]. Several studies have highlighted the antioxidant activity of polyphenols as well as their mechanism of action [94,120,121,122,123]. 

### 4.2. Anti-Inflammatory Effect

According to scientific data, chronic low-grade inflammation is correlated with the development of chronic degenerative diseases, including CVD and diabetes [124,125,126,127]. Resveratrol, a polyphenol found in red wine, grapes [128], or nuts/walnuts [129], is well known for its cardioprotective effects and other beneficial effects, particularly its anti-inflammatory properties. Scientific evidence shows that it can inhibit proinflammatory cytokine production and cyclooxygenase (COX) activity, mediate the NF-κB signaling pathway, endothelial nitric oxide synthetase (eNOS), and inactivate proliferator-activated receptors gamma peroxisomes (PPARγ) [130,131,132,133,134].

Quercetin, another polyphenol with a wide distribution in a multitude of food sources, has the ability to inhibit the biosynthesis of leukotrienes and activate the production of adiponectin, a peptide secreted by adipocytes known for its anti-inflammatory, anti-atherosclerotic, and cardioprotective effects [135,136].

Polyphenols that are found in cocoa (being mostly represented by catechins, proanthocyanidins, and anthocyanins) [137] have been shown to reduce some markers of inflammation, such as interleukins IL-1β and IL-10, adhesion proteins, for example, the adhesion molecule of monocytes-1 (ICAM-1), the adhesion molecule of vascular cells-1 (VCAM-1), monocyte chemotactic protein-1 (MCP-1), or P-selectin, in subjects with high inflammation [138,139].

Scientific evidence for fruit polyphenols shows that they have the ability to significantly reduce markers of inflammation such as C-reactive protein (CRP), IL-6, and tumor necrosis factor (TNF-α) in both healthy subjects and in those with increased cardiometabolic risk [140,141,142]. 

Another study, in which the anti-inflammatory effect of various flavonoid compounds (e.g., quercetin, kaempferol, diosmetin, luteolin, apigenin, genistein, daidzein, and hesperetin) was observed, showed that most of the tested compounds reduced the proliferation induced by macrophages, some of them having the ability to inhibit the production of TNF-α or the Nuclear Factor Kappa B (NF-κB) pathway [143]. Even if the phenolic compounds vary considerably, their anti-inflammatory activity is substantial from a structural point of view [144].

### 4.3. Anti-Atherosclerotic Effect

Atherosclerosis is a progressive inflammatory disease characterized by a reduced vascular lumen due to the accumulation of cholesterol, macrophages, and smooth muscle cells in the form of atheroma plaques that eventually restrict arterial blood flow and reduce blood supply to tissues [145,146].

Endothelial dysfunction is the first step in the development of atherosclerosis and is defined as a reduction in the bioavailability of vasodilators, in particular nitric oxide (NO), and an increase in vasoconstrictors. The vascular endothelium is not just a simple barrier; it is a complex organ, with a role in controlling vascular homeostasis and endothelial dysfunction being correlated with vasoconstriction, thrombosis, increased inflammation, and smooth muscle cell hypertrophy, all of which cause the development of atherosclerotic plaques [147].

Nitric oxide has the role of modulating vascular tone, inhibiting the activation of proinflammatory cytokines, and having antithrombotic and antiapoptotic roles [148,149]. Scientific evidence suggests that dietary polyphenols benefit vascular endothelial integrity by inducing increased NO production and NO expression of endothelial synthase, relaxation, and hyperpolarization mediated by endothelium-derived hyperpolarizing factor [150]. 

Several studies have looked into the protective effects of dietary polyphenols on endothelial cells and blood vessels [112,113,151]. Quercetin looks to have the ability to stimulate tissue plasminogen activator (tPA) and prostacyclin (PGI_2_) release at the level of endothelial cells [152]. Kaempferol has proven its ability to inhibit the conversion of Ang I to Ang II, a beneficial aspect in the treatment of different cardiovascular diseases [153]. In a meta-analysis that aimed to evaluate the relationship between cocoa flavanols and endothelial function, an improvement of flow-mediated dilation (FMD) by 1.08% was highlighted after the consumption of cocoa drink, chocolate, or both for at least 7 days (on average 28 days). Flavanol intake in the analyzed studies ranged between 80 and 1248 mg. It should be noted that a 1% increase in FMD is correlated with a 13% decrease in the risk of cardiovascular events. The optimal effect was registered at an intake of 710 mg total flavanols [112].

Many studies have pointed out that consuming cocoa products, rich in flavanols, determines a decrease in blood pressure, dependent on the daily dose [113,154,155]. In a systematic review, four of eleven studies found statistically significant reductions of ≥5.70 mm Hg in systolic blood pressure (SBP) and ≥2.35 mm Hg in diastolic blood pressure (DBP) after cocoa flavanol consumption [113]. A meta-analysis, including 501 patients, shows that the administration of green coffee extract determined a significant change in SBP, on average (−3.093 mm Hg), and in DBP, on average, (−2.170 mm Hg). The drop in SBP was even more important (−7.21 mm Hg) in the case of a study in which the administered dose was greater than 400 mg of green coffee extract [114]. Even if, at first sight, these changes may seem irrelevant, Tanghe A. et al. remind us that according to scientific data, a 2–5 mm Hg drop in SBP and 2 mm Hg in DBP led to a 3–7% decrease in mortality, a 15% decrease in the risk of stroke or transient ischemic attack, and a 6% decrease in coronary heart disease in the general population aged between 35 and 64 years [113].

Regarding the lipid profile, it is known that an altered lipid profile is correlated with increased cardiovascular risk. While an increased LDL-cholesterol LDL-c value increases cardiovascular risk, an increased HDL-cholesterol HDL-c value confers a protective effect [95].

Studies that followed the effect of supplementing with polyphenols highlighted the reduction of total cholesterol, LDL-C, and increased HDL-cholesterol levels among the subjects [95,156,157].

In a study carried out over a period of 30 days with healthy individuals, the effects of anthocyanin supplementation on cardiovascular risk were monitored. The participants in the study consumed 500 g of fresh strawberries daily after a 10-day strawberry-free period and a diet low in phenolic compounds before the start of the study. Plasma lipid profile was one of the parameters monitored and evaluated, registering a significant reduction in total cholesterol (−8.78%), low-density lipoprotein cholesterol (−13.72%), and triglyceride levels (−20.80%) compared with the baseline period [95].

Another study, this time double-blind, randomized, and placebo-controlled, conducted on 120 subjects with dyslipidemia, aimed to investigate the effects of berry-derived anthocyanin supplementation on the lipid profile in a group of dyslipidemic patients. The anthocyanin group received daily, for 12 weeks, 160 mg of anthocyanins, twice daily. The placebo group received placebo capsules twice daily. Throughout the study, the participants were asked to maintain their usual lifestyle and diet. The study results show that supplementation with anthocyanin increased HDL-cholesterol concentrations by 13.7% compared with 2.8%, an increase achieved in the placebo group. Moreover, there was a decrease in LDL-cholesterol concentrations by 13.6%, compared with −0.6% in the case of the placebo group [95].

Platelet function also plays an important role in the pathogenesis of atherosclerosis, more precisely in the formation of atherosclerotic plaque. Platelet hyperactivation, platelet adhesion, and aggregation are stages in thrombus development [158]. Numerous studies show that polyphenols have the ability to reduce platelet hyperactivity and inhibit platelet aggregation [159,160,161,162].

Consuming fruits rich in polyphenols has been associated with a decrease in the risk of cardiovascular diseases, protective effects against atherosclerosis, and a reduction in premature death and CHD mortality [105,163,164].

A parallel randomized double-blind study, carried out on a group of 200 women during early menopause who consumed a snack bar with a content of 15 g daily between meals of soy protein with 66 mg of isoflavone or only 15 g of soy protein (without isoflavones), was followed by calculating the risk of cardiovascular disease and mortality over 10 years. After 6 months, in the group that consumed the snack bar with a content of 15 g of soy protein and 66 mg of isoflavone, there were very significant results, precisely: a 27% decrease in 10-year coronary heart disease risk, a 24% decrease in cardiovascular disease, a 37% decrease in myocardial infarction risk, and a 42% decrease in cardiovascular disease death risk [105].

### 4.4. Beneficial Effect on Maintaining Glucose and Insulin Homeostasis

Impaired glucose metabolism and insulin resistance are important cardiometabolic risk factors [165]. Several studies have shown that polyphenols have beneficial effects on glucose metabolism and insulin activity through the anti-hyperglycemic effect, inhibiting enzymes responsible for carbohydrate digestion, lowering postprandial blood glucose, modulating glucose transport, increasing glucose absorption in adipose and muscle tissue, protecting pancreatic β-cells against damage, and modulating insulin signaling pathways [166,167,168,169].

A 39–48% reduction in the risk of D2M was observed in a prospective study, for a period of up to 4.6 years, because of polyphenols consumption (flavanones, flavonols and phenolic acids) [170].

Berries, rich in anthocyanins, positively impact postprandial insulin and glucose in prediabetic subjects and overweight and obese adults [171,172,173].

Fallah, A. et al., highlight in a meta-analysis that the intake of dietary anthocyanins determined decreased levels of fasting blood sugar (FBS) by 3.08 mg/dL, two-hour postprandial glucose (2 h PPG) with 16.4 mg/dL of glycosylated hemoglobin (HbA1c), with 0.21%, and the HOMA index (HOMA-IR) with 0.48. The greatest decrease in FBS was recorded among subjects with type 2 diabetes, precisely (−9.28) mg/dL. Important reductions in 2 h PPG were recorded among overweight/obese subjects (−14.6) mg/dL, as well as among those with diabetes (−13.8) mg/dL. HOMA-IR showed the greatest reduction among overweight/obese subjects (−1.80), followed by 1.08 in the case of those with diabetes. It is also emphasized that a duration greater than 8 weeks of the intervention and a dose of anthocyanins higher than 300 mg/day were more efficient in improving the glycemic profile [106].

### 4.5. Prebiotic Effect on the Gut Microbiota

Recent studies suggest that polyphenols have a prebiotic effect on the gut microbiota by stimulating the activity of microbial species such as *Lactobacillus, Bifidobacterium, Faecalibacterium, Akkermansia*, and *Roseburia* and the production of SCFA [174,175]. When used as a prebiotic substrate, polyphenols promote the growth and colonization of probiotic bacterial families like *Lactobacillaceae* and *Bifidobacteriaceae* while simultaneously lowering the prevalence of pathogenic bacteria like *Clostridium perfringens*, *Escherichia coli*, and *Helicobacter pylori* [176,177], process involving changes in the resistance and permeability of the bacterial membrane through changes in the amount of favorable and pathogenic bacteria, suggesting an alteration in the structure of short-chain fatty acids (SCFAs), along with a decline in obesity incidence and inflammatory processes [178]. Strong evidence in this regard is available from studies with both non-flavonoid and flavonoid polyphenols, especially based on preclinical studies, but also clinical trials [174]. The prebiotic effect is associated with many health benefits, including reduced systemic inflammation, increased insulin sensitivity, an improved lipid profile, improved intestinal function, and the absorption of minerals [179,180,181,182,183]. 

A clinical trial, double-blind, crossover, placebo-controlled, conducted on a group of 49 overweight-obese subjects with mild hyperlipidemia for nine weeks, followed the effect of consuming pomegranate extract on the gut microbiota. The results showed the increase of important microorganisms in maintaining homeostasis at the level of the gut microbiota, specifically *Faecalibacterium*, *Bacteroides, Odoribacter, Butyricicoccus,* and *Butyricimonas*. There was also a reduction in microorganisms with inflammatory potential, such as *Parvimonas* and *Methanosphaera* but also in the plasma concentration of lipopolysaccharide-binding protein [181].

Supplementing with polyphenols increases the number of *Lactobacillus* and *Bifidobacterium*, inhibiting at the same time the abundance of *Clostridium* pathogenic species, as demonstrated by in vitro and in vivo human research [184,185]. The growth percentage recorded in the case of *Lactobacillus* was up to 220%, the most effective medium dose intake in this direction, proving to be 396 mg/d for in vivo studies and <62.5 mg/L for in vitro studies. In the case of *Bifidobacterium*, supplementing the intake of polyphenols brought an increase in abundance a 56%, with a dose of 540 mg/d appearing to be the most effective for in vivo studies and 23 mg/L for in vitro studies. An important aspect to note is the one related to the dose; more precisely, at a dose higher than 396 mg/day, the stimulatory effect on *Lactobacillus* decreased, and at more than 593 mg/day, it no longer existed. Regarding *Bifidobacterium*, an intake of <540 mg/d brought marginal results, while at an intake of >540 mg/d, the abundance-stimulating effects began to diminish. Regarding the source of polyphenols, in in vivo studies, the best results in stimulating the growth of *Lactobacillus* were recorded with blueberry intake, followed by red wine intake. In the *Bifidobacterium* case, the intake of apples brought them the best results, followed by the intake of red wine [184,186].

## 5. Crosstalk between Dietary Polyphenols and Gut Microbiota

It is thought that polyphenols and the gut microbiome interact reciprocally. It is known that only 5–10% of ingested polyphenols are absorbed in the small intestine due to glycosidic bonds, which limit their absorption at this level. The rest reach the large intestine, where they have the ability to modulate the abundance of certain microorganisms [187]. However, the activity is not unidirectional, with the gut microbiota having the ability to convert polyphenols into more active and better-absorbed metabolites. Therefore, both native polyphenols and their metabolites participate in maintaining the intestinal homeostasis of the host by becoming a substrate for probiotics and determining the growth of beneficial bacteria and the reduction of pathogenic bacteria [47,188]. Furthermore, microbiota strongly connects metabolic and chronic disorders with the health-promoting effects of polyphenols.

Phenolic structure, flavonoid or nonflavonoid factors, spatial configuration, and polymerization degree all influence the microbial transformations [47,189].

Polyphenols belonging to the flavonoid group (flavonols, flavones, flavanones, flavanols, isoflavones, and anthocyanins) all have in their structure two benzene rings linked by a heterocyclic pyrone C-ring, existing in foods, and they are found as glycosides, C-glycosides, and O-glycosides, flavan-3-ols not conjugated [190].

Among the flavonols, the most popular is quercetin, which, although it has a low solubility and reduced stability in the upper gastrointestinal tract, therefore a limited bioavailability, has been intensively studied due to the benefits observed in order to prevent cardiovascular diseases, diabetes, and more. Present in apples, berries, onions, tomatoes, tea, and red wine, quercetin is metabolized in the colon by luminal bacteria (*Eubacterium oxidoreducens*, *Eubacterium ramulus*, *Clostridium orbiscidens*, and *Enterococcus casseliflavus*) to a series of phenolic acids: 3-hydroxyphenylacetic acid, 3,4-dihydroxyphenylacetic acid, 4-hydroxyphenylacetic acid, and hippuric acid. Quercetin, along with the mentioned metabolites, has proven anti-inflammatory and antioxidant effects, normalizing the lipid profile by reducing triglycerides, total cholesterol, and LDL cholesterol, respectively, and having anti-diabetic properties by maintaining glucose homeostasis and improving insulin resistance [32,187,191].

A study conducted in Japan followed the effects of apple consumption on the gut microbiota of human subjects. Participants in the study, men between the ages of 21 and 60, followed a normal diet without any restrictions one week before the experiment and one week after. During the two weeks the experiment lasted, the participants consumed two apples daily. The participants were not given any medication or food containing viable cultures for four weeks before and during the investigation. Fecal samples taken (days 0, 7, and 14 during consumption and day 7 after consumption) revealed an increase in *Bifidobacteria*, *Lactobacillus*, and *Streptococcus*, respectively, and a decrease in *Pseudomonas*, *C. perfringens*, and *Enterobacteriaceae* [192].

Flavones, represented by apigenin and luteolin, are present in the peel of citrus fruits, in parsley and celery, or in cereals in the form of glycosidic derivatives, which at the intestinal level are hydrolyzed by digestive enzymes, and the unabsorbed aglycones will be metabolized in the large intestine by microorganisms such as *Enterococcus avium* or *Clostridium orbiscinde*. Some of the metabolites of apigenin are: 3-(3-hydroxyphenyl)-propionic acid, 3-(4-hydroxyphenyl)-propionic acid, 4-hydroxycinnamic acid, and phloretin chalcone [32,187].

Flavanones, represented by naringenin or hesperetin, are present especially in citrus fruits and tomatoes and have a higher bioavailability than flavan-3-ols or flavonols. Deglycosylation and metabolism at the gut microbiota level are similar to flavonols, mainly under the action of *Eubacterium ramulus* and *Clostridium* species, respectively. The most representative metabolites of naringenin are: 3-(4-hydroxyphenyl)-propionic acid, 3-(3,4-dihydroxyphenyl)-propionic acid, and 3-(3-hydroxyphenyl)-propionic acid [32,187]. Over time, following various in vivo or in vitro studies, antioxidant, anti-adipogenic, anti-inflammatory, and cardioprotective activities have been attributed to naringenin [193].

Flavanols (flavan-3-ols) are represented by catechin, epicatechin, epigallocatechin, or proanthocyanidins and are found in foods such as berries, apples, eggplant, red onions, nuts, black and green tea, red grapes, red wine, cocoa, or dark chocolate [32]. In the colon, especially under the action of *Bifidobacterium* spp., *Clostridium coccoides*, *Flavonifractor plautii*, and *Eggerthella lenta*, their metabolites result, the most important of which being hydroxy-phenyl-γ-valerolactones and hydroxy-phenylvaleric acids, but also derivatives of phenylacetic, phenylpropanoic, and benzoic acid, common end products of polyphenol catabolism in the colon. The specialized literature shows several functions of these metabolites, including the antioxidant and anti-inflammatory effects, the decrease in NO synthesis and iNOS expression, the reduction of systolic blood pressure, and the positive influence on endothelial function [187]. Lee et al., for example, reported that monocyte-endothelial cell adhesion was prevented after treatment with concentrations under 30 µM of 5-(3′,4′-dihydroxyphenyl)-γ-valerolactone in a study carried out on human cells [194]. In an in vivo human study, the subjects consumed drinks with high (494 mg cocoa flavanols/d) and low (23 mg cocoa flavanols/d) quantities for four weeks each, with a four-week washout period between. Fecal samples were collected before and after each intervention, observing, in the case of the group with high consumption of cocoa flavanols, a significant increase of *Bifidobacteria* and *Lactobacillus* but also of *Eubacterium rectale* and, respectively, a decrease of *Clostridium* [192].

Tea flavonoids were demonstrated to relieve atherosclerosis by reducing serum TMA through gut microbiota modulation [195]. Moreover, the intake of tea polyphenols in the diet caused a decrease in *Bacteroides,* an increase in *Firmicutes*, and a rise in the *Firmicutes* to *Bacteroides* ratio in feces [196]. Incorporating almonds, almond skin, pistachios, or other nuts in the diet can enhance the amount of *Lactobacillus* and *Bifidobacteria* in feces [197,198].

Isoflavones, represented by genistein or dadzein, are, as is well known, phytoestrogen precursors, being present in soy and produced from soy, respectively, in legumes [32,199]. Transformed by intestinal enzymes and under the action of gut microbiota, especially *Bacteroides ovatus*, *Enterococcus faecium*, *Lactobacillus mucosae*, *Ruminococcus productus, Streptococcus intermedius*, *Finegoldia magna*, and *Clostridium* spp., into active metabolites with important estrogenic activity, such as 4’,7-isoflavandiol (equol), or inactive compounds from this point of view, such as O-desmethylangolensin [187]. The anti-atherogenic and cardioprotective effects and stimulation of the antioxidant systems at the cellular level have been reported in their case [200,201].

Anthocyanins, represented by cyanidin, delphinidin, malvidin, peonidin, or petunidin, are found in berries, mango, oranges, red onions, olives, cabbage, beans, and red wine [32]. Protocatechuic acid, gallic acid, 4-Hydroxybenzoic acid, vanillic acid, and syringic acid are the bioactive metabolites that are produced under the action of *Lactobacillus plantarum, Lactobacillus casei*, *Lactobacillus acidophilus*, *Lactobacillus acidophilus*, and *Bifidobacterium lactis* [187]. An important source of anthocyanins, but also proanthocyanidins, flavonols, and phenolic acids, are elderberries, which have proven multiple beneficial effects, including cardiovascular protection, antidiabetic properties, and a prebiotic effect. A recent study revealed the prebiotic potential of elderberries on *Lactobacillus plantarum*, *Lactobacillus casei*, *Lactobacillus rhamnosus*, *Lactobacillus fermentum*, and *Saccharomyces boulardii.* The highest increase (*p* < 0.05) was recorded at 1.5% freeze-dried elderberry powder on *Lactobacillus casei* with a 152.44% growth index. The same study highlighted a significant bioaccessibility index of elderberry polyphenols during simulated digestion (75%), this aspect being essential for expected effects [202]. Anthocyanins, which are also found in abundance in blueberries, can increase the quantity of lactic acid bacteria and *Bifidobacteria* in healthy individuals [203].

Non-flavonoid phenolic compounds have a higher degree of polymerization and are more heterogeneous in structure than flavonoid ones [47].

The most structurally complex in this class are the hydrolysable tannins, with the best-known representatives being ellagitannins and gallo tannins, especially found in berries, guava, and nuts [204]. The ellagitannins that reach the colon are hydrolyzed mainly to ellagic acid and urolithin (A and B being the most significant in concentration and physiological activity) under the action of *Butyrivibrio* spp. The type of urolithin produced closely depends on the colon’s microbiome. Furthermore, ellagic acid will be transformed under the action of intestinal microbiota, through dehydroxylation, into urolithins [47,138].

Urolithin A has been shown to have beneficial effects on the heart. Recent data claim that it reduces inflammation after cardiovascular injury and confers endothelial protection by normalizing NO production and reducing inflammation [205]. Urolithin B has recently attracted attention due to its nutraceutical potential. According to the results of several studies, its potent anti-inflammatory and antioxidant effects can become an essential ally in preventing cardiovascular diseases and diabetes in the fight against hyperlipidemia and cancer [206,207].

Additionally, high intake of polyphenols help control the body’s *Firmicutes* to *Bacteroides* ratio. Proanthocyanidin-rich cranberries can decrease the number of *Firmicutes* and raise the amount of *Bacteroides* in the body when consumed regularly, an aspect observed in both animal and human gut microbiota. In addition to this aspect, phenolic compounds from cranberries seem to stimulate the growth of *Akkermansia* spp., respectively, to reduce *Clostridia* and *Oribacterium* [28,192,208,209]. 

Resveratrol is the best-known representative of stilbenes. It is present in grapes and red wine, mainly because it is easily absorbed at the intestinal level and quickly metabolized, resulting in sulfate and glucuronide conjugates. Then, under the action of gut microbiota, especially *Adlercreutzia equolifacie* and *Slackia equolifaciens*, it is transformed into dihydroresveratrol, respectively 3,4’-dihydroxytrans-stilbene and 3,4′-dihydroxybibenzyl [187]. Resveratrol is “famous” for its cardio-protective effects due to its antioxidant, anti-inflammatory, and antithrombotic activity, improving vascular function, inhibiting apoptosis, and delaying atherosclerosis. Some studies highlight the beneficial effects of resveratrol in MetS in people with obesity, diabetes, or insulin resistance [210].

Polyphenols from red wine may also help reduce the cardiovascular and metabolic changes brought on by obesity. Dealcoholized red wine and red wine (272 mL per day) were administered to Mets (patients with metabolic syndrome) or healthy volunteers over a period of 30 days in Moreno-Indias and colleagues’ study. The indicators of metabolic syndrome, such as triglycerides, glucose, and total cholesterol, ameliorated after drinking dealcoholized red wine and red wine. In the case of the Mets group, triglycerides decreased on average by 32%, total cholesterol by 37.8%, blood sugar by 25%, and CRP by 38.9%. Positive differences were also recorded in the healthy group. *Proteobacteria* and *Firmicutes* are more prevalent in Mets participants than in healthy participants, according to a PCR-DGGE analysis of the fecal microbiota. However, after the intake of dealcoholized wine and wine, no significant variations were seen in the microbiome composition of the two volunteer groups. Following wine intake, the levels of *Bacteroidetes, Fusobacterium*, *Faecalibacterium prausnitzii*, *Lactobacillus*, and *Roseburia* (a butyrate-producing bacteria) were higher, whereas the levels of *Clostridium*, *Firmicutes*, and *Clostridium histolyticum* were lowered in the Mets participants [211].

Additionally, grape pomace, a byproduct of making wine that contains polyphenols and dietary fiber, can benefit the intestinal dysbiosis of people with metabolic syndrome. Participants who presented cardiometabolic risk (those with a minimum of two metabolic syndrome indicators) received an 8 g daily dose of grape pomace as a supplement for six weeks. Only half of the subjects responded to the treatment with grape pomace, with their insulin levels being reduced by at least 10% compared with those in the control group, in which supplementation was not performed and whose insulin values were increasing. Pomace also tended to decrease the representatives of the order *Lactobacillales* in both non-responders and responders, yet to raise *Bacteroides* only in non-responders [212].

Resveratrol and other polyphenols were demonstrated to reduce circulatory trimethylamine-N-oxide TMAO by controlling its synthesis through gut microbiota remodeling [213], with a recent RCT on humans supporting these findings [214]. 

According to Queipo-Ortu, the combination of alcohol and polyphenols increased the amount of *Bacteroides, Bacteroides uniformis*, *Bifidobacterium*, *Enterococcus, Eggerthella lenta*, *Blautia coccoides–Eubacterium*, and *Prevotella*, although these substances had no considerable impact on the adjustments of Lactobacillus [215].

Phenolic acids, represented by cinnamic (caffeic acid, p-coumaric acid, and ferulic acid) and benzoic acid derivatives (p-hydroxybenzoic acid, gallic acid, and ellagic acid), are present in sources such as whole grains, coffee, red wine, vegetables, red fruits, berries, and spices. The final metabolites in the case of cinnamic derivatives are hydroxybenzenes, resulting from decarboxylation. Hydroxybenzoic acids are the most widespread microbial metabolites resulting from the activity of the intestinal microbiota on phenolic compounds, both flavonoid and non-flavonoid types [47].

Researchers have suggested that foods like artichokes, apples, chocolate, and strawberries that contain necessary hydrocaffeic acid precursors may reduce intestinal inflammation and support anti-inflammatory activity. As a consequence of polyphenol breakdown, Tucsek et al. discovered that, for instance, ferul aldehyde can be used to reduce mitogen-enacted protein kinase (MAPK) expression, suppressing NF-βB action, the production of reactive oxygen species (ROS), and mitochondrial depolarization [216]. 

According to Beloborodova et al., p-hydroxyphenyl acetic acid can prevent neutrophils from generating reactive oxygen species (ROS), and phenolic acids can serve as indicators of sepsis [217].

The gut microbiota can also transform lignans, resulting from several reduction, dehydroxylation, demethylation, and lactonization reactions, into enterodiol and enterolactone, two mammalian phytoestrogens. The main microorganisms responsible for bacterial catabolism for lignans are *Bacteroides fragilis*, *Bacteroides ovatus*, *Bacteroides distasonis*, *Clostridium cocleatum*, *Clostridium scindens*, *Butyribacterium methylotrophicum*, *Eubacterium limosum*, *Eubacterium callanderi*, and *Peptostreptococcus productus* [187].

There is a connection between obesity and gut dysbiosis. In addition, obesity is a key contributor to the risk of metabolic disorders such as cardiovascular impairment, fatty liver disease, and diabetes. Polyphenols can reduce dysbiosis by decreasing systemic inflammation, as previously described, and diets high in polyphenols may provide the same benefits. In a 12 week investigation of obese women, juice from *Schisandra chinensis* fruits was consumed twice daily in doses of 100 mL each. According to the findings, *Schisandra chinensis*, an important source of lignans, reduced alanine aminotransferase, aspartate aminotransferase, triglycerides, fasting blood sugar levels, and fat mass. Denaturing gradient gel electrophoresis (DGGE) examination of the gut microbiome revealed that while the amount of *Ruminococcus* was lower in the treatment group compared with the placebo group, the amount of *Akkermansia*, *Bacteroides*, *Bifidobacterium*, *Prevotella*, and *Roseburia* increased [218].

Some in vivo human studies that explore dietary polyphenols and gut microbiota interactions are summarized in Table 2.

There is, therefore, a wide range of evidence supporting the benefits of polyphenols on health, both in the prevention and management of certain chronic diseases. Beyond these benefits and mechanisms of action, other very valuable aspects can make a difference in the results and safety. Some of these aspects are the form of the phenolic compound, the dose, and the potential unwanted effects. Regarding the dose, it is important to remember what Paracelsus said: “*All things are poison, and nothing is without poison; only the dose makes a thing not a poison*.” In the case of polyphenols, a potential toxicity level can appear due to an intake of megadoses from supplements or overly fortified foods [225]. In the case of dietary supplements, potential interactions and toxicity are not thoroughly evaluated, as are drugs. Overdose can easily occur because polyphenol supplements contain high doses of pure polyphenols and are easily accessible, both in terms of availability and price. A recent publication draws attention to the fact that, for example, to obtain 1 g of quercetin, found in a capsule of a food supplement and which you swallow quickly, a person would have to consume 2.4 kg of dried oregano (the food source with the highest concentration), which is obviously impossible [226]. Human studies that pay more attention to these aspects too, or experimental studies that simulate human physiology as faithfully as possible, are a continuous necessity. Also, some dietary reference intake regulation would be very useful.

## 6. Conclusions

A diet high in vegetables and fruits is linked to a lower risk of various lifestyle-related nontransmissible illnesses, besides CVD. Numerous necessary and functional micronutrients, including polyphenols, are provided by fruits and vegetables. Because of their importance for bioavailability and human health, interactions between dietary components, particularly phenolic substances, and gut bacteria have received a lot of interest. Besides their primary roles as antioxidants and anti-inflammatory agents, polyphenols also have the ability to modulate the gut microbiota. Several studies have demonstrated the interaction between gut microbiota and polyphenols in both in vitro and in vivo conditions. These substances serve as the basis for probiotics due to their structure and the metabolites they produce, which encourage the growth of good bacteria while suppressing the growth of pathogenic ones, preserving the intestinal homeostasis of the organism.

It is yet unclear if dietary polyphenols, either alone or in combination, would treat cardiovascular failure directly or indirectly by regulating the gut microbiota. However, in vivo and in vitro results show their clinical potential and preventive and therapeutic impact. Further experimental and reliable information sustained by clinical research is required to establish polyphenols’ positive effects on gut symbiosis, antibacterial effects on pathogenic microorganisms, prebiotic effects, and benefits on the cardiovascular system.

## Figures and Tables

**Table 1:** Aspects of the gut microbiota in subjects with cardiometabolic risk.

Cardiometabolic Risk Components	Special Aspects of the Gut Microbiota	Refs.
Hypertension	↑ *Firmicutes*/*Bacteroides* ratio, ↓ bacterial diversity ↑ *Klebsiella*, *Prevotella*, *Desulfovibrio*, *Clostridium*, *Oscillibacter* ↓ *Faecalibacterium*, *Akkermansia*, *Roseburia*, *Butyrivibrio*, *Lactobacillus* ↓SCFA	[57,64]
Heart failure	↓ *Firmicutes*/*Bacteroides* ratio, ↓ bacterial diversity ↑ *Escherichia*, *Klebsiella*, *Streptococcus*, *Rumnicoccus* ↓ *Faecalibacterium*, *Eubacterium*	[71,72]
Atherosclerosis	↓ bacterial diversity ↑ *Streptococcus*, *Pseudomonas*, *Klebsiella*, *Veillonella* spp., ↓ *Roseburia*, *Faecalibacterium*, *Bacteroides* spp. ↑ TMAO, ↓ SCFA	[61,73]
T2DM and obesity	↑ *Firmicutes*/*Bacteroides* ratio, ↓bacterial diversity ↑ *Lactobacillus reuteri*, *Proteobacteria* ↓ *Faecalibacterium*, *Akkermansia*, *Roseburia*, *Methanobrevibacter smithii* ↓ SCFA, ↑ LPS	[56,60,61,62,63,64]

↑—high, ↓—low, SCFA—short-chain fatty acids, TMAO—trimethylamine N-oxide, LPS—lipopolysaccharides.

**Table 2:** Dietary polyphenols and gut microbiota interactions.

Polyphenol Source	Method	Outcome	Ref.
Blackcurrant	Clinical trial (30 healthy human volunteers): consumption of blackcurrant extract First Leaf 1500 mg/day and Cassis Anthomix 672 mg/day for 4 week and 2-week washout.	↑ *Lactobacillus* and *Bifidobacteria* ↓ *Clostridium* spp. and *Bacteroides* spp.	[219]
Elderberry	Clinical trial (30 healthy human volunteers): consumption of purified extract from European black elderberries with a high and standardized content of polyphenols and anthocyanins (600 mg/day). Nine week, three distinct phases, three weeks each (baseline, intervention, and washout).	↑ *Akkermansia* spp. and *Bacteroides cellulosolyticus*	[220]
Blueberry	Double-blind randomized clinical study (20 healthy male individuals): consumption of a wild blueberry drink (25 g of wild blueberry powder in 250 mL of water) every day for 6 weeks.	*↑ Bifidobacterium* spp.	[203]
Cocoa	Randomized, controlled, double-blind, crossover intervention (22 healthy human volunteers): consumption of cocoa flavanols (high quantity–494 mg cocoa flavanols/d, and low quantity–23 mg cocoa flavanols/d) for 4 weeks each, with a 4 week washout period.	↑ *Bifidobacteria* and *Lactobacillus* ↓ *Clostridium* spp., triacylglycerol, and C-reactive protein concentrations	[185]
	Randomized clinical trial (30 moderately obese with BMI between 30 and 35 kg/m^2^) consumption of 10 g fark chocolate (4 weeks).	↑ *Lactobacillus* ↓ *Bacteroidetes*	[221]
Almond	Randomized, crossover-controlled intervention (48 healthy subjects): almond (56 g) and almond skin (10 g) ingestion, with commercial fructo-oligosaccharides (8 g) as positive control (6 weeks).	↑ *Bifidobacterium* spp., *Lactobacillus* spp. and fecal β-galactosidase activity ↓ *Clostridum perfringens*, fecal β-glucuronidase, nitro reductase and azo-reductase activities	[197]
Green tea	Randomized, crossover-controlled intervention (12 healthy volunteers): green tea liquid consumption (400 mL/day 2 weeks).	↑ *Firmicutes: Bacteroidetes* ratio, SCFA producing genera ↓ bacterial LPS synthesis	[196]
Red wine polyphenols	Randomized, crossover-controlled intervention (10 metabolic syndrome patients and 10 healthy subjects): consumption of red wine or dealcoholized red wine (272 mL per day), 30 days each after a washout period.	In the metabolic syndrome patients, ↑ *Bifidobacteria*, *Lactobacillus* and butyrate-producing bacteria (*Faecalibacterium prausnitzii* and *Roseburia*) ↓ LPS producers (*Escherichia coli* and *Enterobacter cloacae*)	[211]
	Randomized, crossover, controlled intervention (10 healthy male volunteers): consumption of red wine (272 mL/d) and dealcoholized red wine (272 mL/d) and gin (100 mL/d), 20 days each after a washout period.	↑ *Enterococcus*, *Bifidobacterium*, *Bacteroides*, *Prevotella*, *Bacteroides uniformis*, *Eggerthella lenta*, and *Blautia coccoides*. ↓ blood pressures, total cholesterol, triglyceride, and C-reactive protein concentrations	[215]
Pomegranate	Double-blind, cross-over, dose-response, randomized, and placebo controlled clinical trial (49 overweight obese subjects with mild hyperlipidemia): consumption two doses (D1 = 164 mg and D2 = 656 mg lasting 3 weeks each) of pomegranate extract or placebo alternating with 3 weeks of washout periods.	↑ *Bacteroides*, *Faecalibacterium*, *Butyricicoccus*, *Odoribacter*, and *Bacteroides*, *Faecalibacterium*, *Butyricicoccus*, *Odoribacter*, and *Butyricimonas*. ↓ *Parvimonas*, *Methanobrevibacter*, and *Methanosphaera*. ↓ high-sensitivity C-reactive protein and lipopolysaccharide-binding protein	[181]
Orange	Randomized crossover design (21 healthy volunteers): 500 mL of orange juice from *Citrus sinensis* L. Osbeck cv. Cara Cara, Bahia, or isocaloric control drink. Seven days with a one-week washout period between consecutive interventions.	↑ *Mogibacteriaceae*, *Tissierellaceae*, *Veillonellaceae*, *Odoribacteraceae*, and *Ruminococcaceae*	[222]
Phenolic acids, Dietary raisin	Exploratory feeding study (13 healthy volunteers): consumption of three servings (28.3 g each) of sun-dried raisins daily for 14 days (on days 7 and 14).	↑ *Faecalibacterium prausnitzii*, *Bacteroidetes* spp., *Ruminococcus* spp.; *Decrease Klebsiella* spp., *Prevotella* spp., *Bifidobacterium* spp.	[223]
Aronia berry	Double-blind randomized controlled trial (66 healthy men): consumption of (poly)phenol-rich extract (116 mg, 75 g berries), a whole fruit powder (12 mg, 10 g berries), or placebo (maltodextrin) for 12 wk.	extract ↑ *Anaerostipes* whole fruit *Bacteroides*	[224]
Grape pomace	Randomized cross-over clinical trial (49 subjects at cardiometabolic risk exhibiting at least two metabolic syndrome factors): consumption of a daily dose of 8 g of grape pomace for 6 weeks with an equivalent control period.	↓ *Lactobacilliales* and Insulin in responders	[212]

↑—high, ↓—low, SCFA—short-chain fatty acids, LPS—lipopolysaccharides.
